# Supplementary material for: The prognostic and diagnostic value of intraleukocytic malaria pigment in patients with severe falciparum malaria
Source: Nat Commun. 2022 Nov 12;13:6882. doi: 10.1038/s41467-022-34678-8 (PMC9653500; doi:10.1038/s41467-022-34678-8)
Supplement: Supplementary file 1 — Supplementary Materials [file 41467_2022_34678_MOESM1_ESM.pdf]

Supplementary Materials: "The prognostic and diagnostic value of intraleukocytic malaria pigment in patients with severe falciparum malaria"

Ketsanee Srinamon, James A Watson, Kamolrat Silamut, *et al.*

October 28, 2022

Supplementary Tables and Figures

Table S1: Pooled individual patient data set. The median (range) age is shown. n is the total number of patients enrolled in each study.

Study	Countries	Years	n	Deaths	Age (years)	Coma	PMN counts	PMM counts	Parasite counts
AQ Vietnam [1]	Vietnam	1991-1996	560	83 (15%)	30 (15-79)	52%	483	301	560
SEAQUAMAT [2]	Myanmar, India, Bangladesh, Indonesia	2003-2005	1461	270 (19%)	25 (2-87)	43%	1330	1329	1457
AQUAMAT [3]	Mozambique, The Gambia, DRC, Tanzania, Kenya, Rwanda, Nigeria, Ghana, Uganda	2005-2010	5425	527 (10%)	2 (0-14)	36%	4211	4186	4786
SMAC [4]	The Gambia, Kenya, Malawi, Ghana, Gabon	2000-2005	26389	1136 (4%)	2.2 (0-15)	9%	25845	25025	26199
Lyke *et al.* [5]	Mali	2000-2001	172	15 (9%)	2.5 (0.2-10.4)	21%	166	166	172

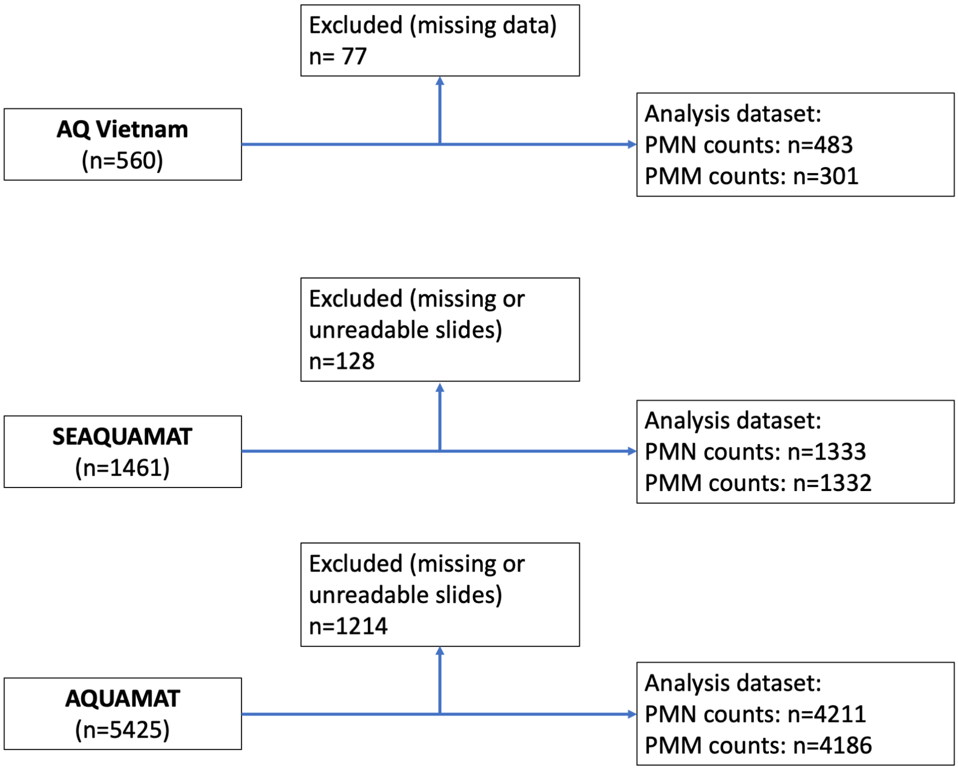

Figure S1: Flow diagram for inclusion and exclusion of data from the three randomised trials.

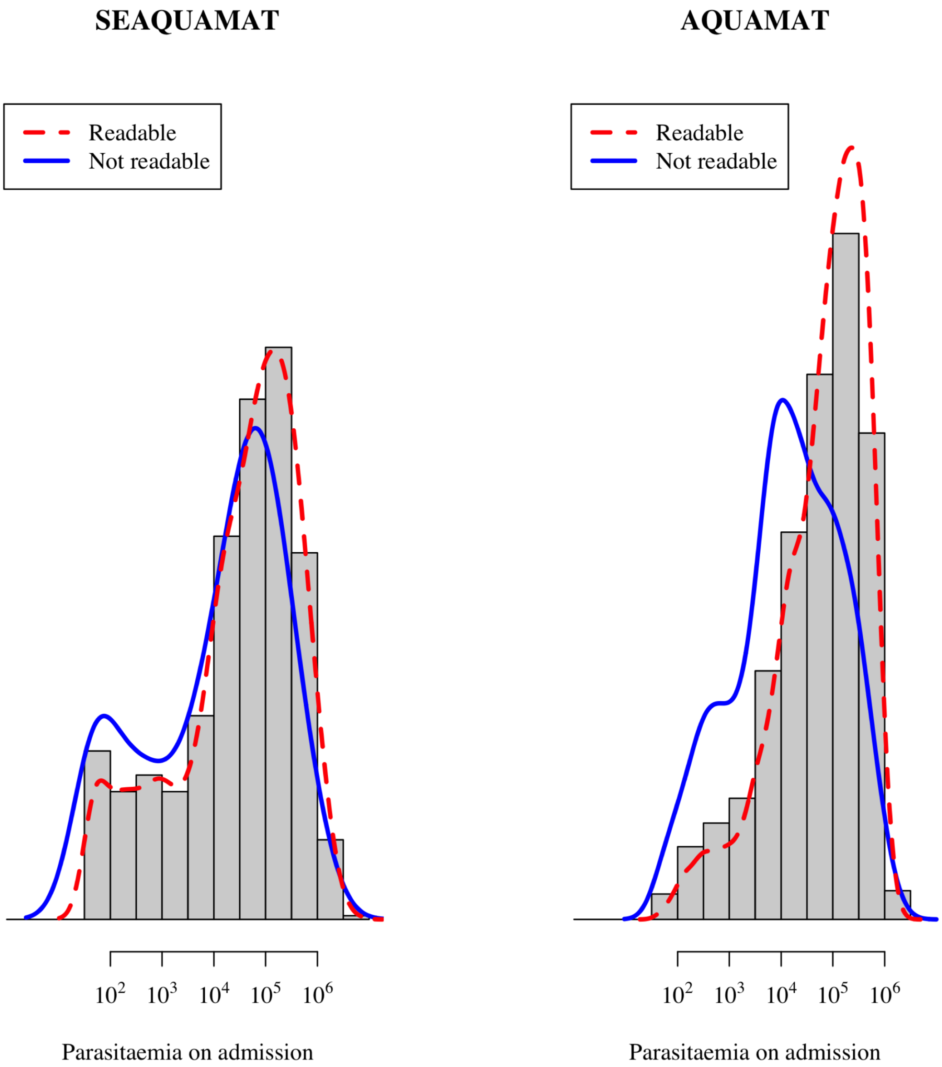

Figure S2: Relationship between malaria parasitaemia and data availability on pigment counts in the SEAQUAMAT and AQUAMAT randomised trials.

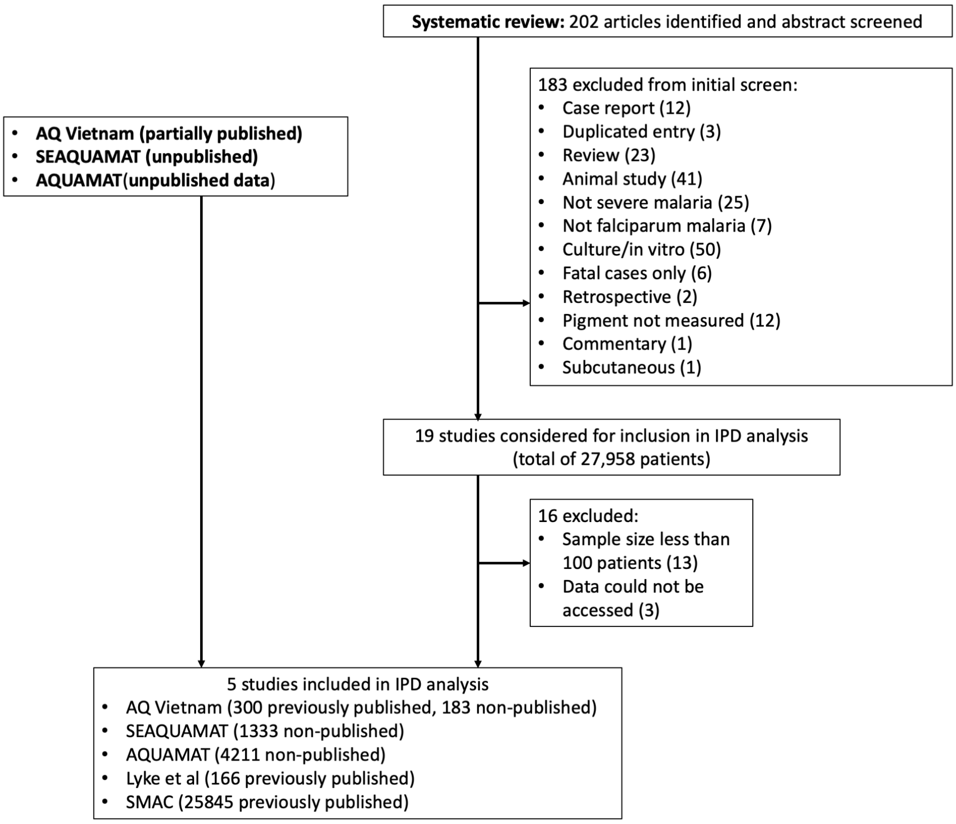

Figure S3: Flow diagram for inclusion and exclusion of data following the systematic review.

Study	>5% pPMM Events	>5% pPMM Total	<5% pPMM Events	<5% pPMM Total	OR
AQ: Vietnam	37	218	3	83	5.45
SEAQUAMAT: Bangladesh	32	125	82	285	0.85
SEAQUAMAT: Myanmar	40	197	42	356	1.90
SEAQUAMAT: Indonesia	7	23	17	212	5.02
SEAQUAMAT: India	20	49	12	85	4.20
AQUAMAT: Mozambique	18	185	18	209	1.14
AQUAMAT: DRC	12	293	3	87	1.20
AQUAMAT: Ghana	6	199	6	95	0.46
AQUAMAT: Nigeria	19	172	12	110	1.01
AQUAMAT: Kenya	28	208	8	93	1.65
AQUAMAT: Tanzania	112	742	59	465	1.22
AQUAMAT: Rwanda	6	100	6	105	1.05
AQUAMAT: The Gambia	30	267	21	219	1.19
AQUAMAT: Uganda	12	287	23	350	0.62
SMAC: The Gambia	152	1165	174	2278	1.81
SMAC: Ghana	84	1725	227	5071	1.09
SMAC: Kenya	43	1192	202	5604	1.00
SMAC: Gabon (Lambarene)	7	288	19	1523	1.97
SMAC: Gabon (Libreville)	11	203	38	703	1.00
SMAC: Malawi	22	492	113	4781	1.93
Lyke et al: Mali	10	109	5	57	1.05
**Fixed effect model**		**8239**		**22771**	**1.35**
**Random effects model**					**1.37**

Heterogeneity: $I^2 = 60\%$, $\tau^2 = 0.0809$

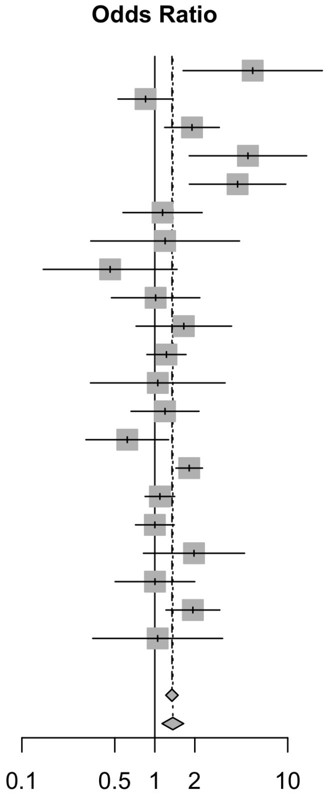

Figure S4: Meta-analysis of the prognostic value for mortality of >5% versus ≤ 5% pigment containing monocytes in 31,007 patients clinically diagnosed with severe falciparum malaria. The individual estimates (vertical ticks) and 95% confidence intervals (horizontal lines) are shown for each study broken down by country except for SMAC, Gabon where the distribution of pigment counts was substantially different between the two sites (Lambaréné and Libreville).

Study	>5% pPMN Events	>5% pPMN Total	<5% pPMN Events	<5% pPMN Total	OR
AQ: Vietnam	33	92	32	383	6.14
SEAQUAMAT: Bangladesh	54	160	50	220	1.73
SEAQUAMAT: Myanmar	52	185	22	269	4.39
SEAQUAMAT: Indonesia	9	25	11	138	6.49
SEAQUAMAT: India	16	52	15	78	1.87
**Fixed effect model**		**514**		**1088**	**3.24**
**Random effects model**					**3.44**

Heterogeneity: I^2 = 77%, τ^2 = 0.2118

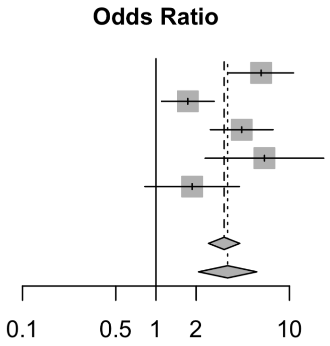

Figure S5: Pooled data set: prognostic value for mortality of >5% versus ≤ 5% pigment containing PMNs (neutrophils) in adults (defined as >15 years). The individual estimates (vertical ticks) and 95% confidence intervals (horizontal lines) are shown for each study broken down by country. None of the patients in the African studies were over 15 years of age so this only includes data from Asia.

Study	>5% pPMN Events	>5% pPMN Total	<5% pPMN Events	<5% pPMN Total	Odds Ratio	OR
AQ: Vietnam	0	1	1	7		1.44
SEAQUAMAT: Bangladesh	6	11	4	19		4.50
SEAQUAMAT: Myanmar	4	41	4	58		1.46
SEAQUAMAT: Indonesia	2	7	2	66		12.80
SEAQUAMAT: India	1	2	0	2		5.00
AQUAMAT: Mozambique	11	89	27	310		1.48
AQUAMAT: DRC	8	66	7	309		5.95
AQUAMAT: Ghana	4	101	8	194		0.96
AQUAMAT: Nigeria	13	66	18	218		2.73
AQUAMAT: Kenya	26	143	10	160		3.33
AQUAMAT: Tanzania	71	389	98	816		1.64
AQUAMAT: Rwanda	5	35	7	170		3.88
AQUAMAT: The Gambia	15	101	36	385		1.69
AQUAMAT: Uganda	6	90	30	547		1.23
SMAC: The Gambia	21	97	304	3342		2.76
SMAC: Ghana	73	686	238	6102		2.93
SMAC: Kenya	31	598	214	6195		1.53
SMAC: Gabon (Lambarene)	7	292	19	1514		1.93
SMAC: Gabon (Libreville)	13	109	75	1616		2.78
SMAC: Malawi	11	57	124	5198		9.79
Lyke et al: Mali	6	41	9	125		2.21
**Fixed effect model**		**3022**		**27353**		**2.26**
**Random effects model**						**2.37**

Heterogeneity: $I^2 = 55\%$, $\tau^2 = 0.0712$

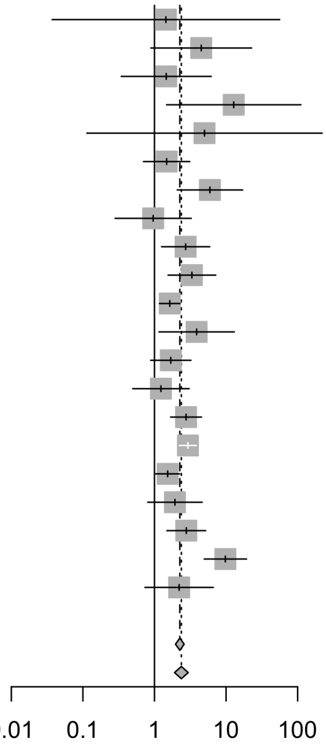

Figure S6: Pooled data set: prognostic value for mortality of >5% versus ≤ 5% pigment containing PMNs (neutrophils) in children (defined as ≤15 years). The individual estimates (vertical ticks) and 95% confidence intervals (horizontal lines) are shown for each study broken down by country.

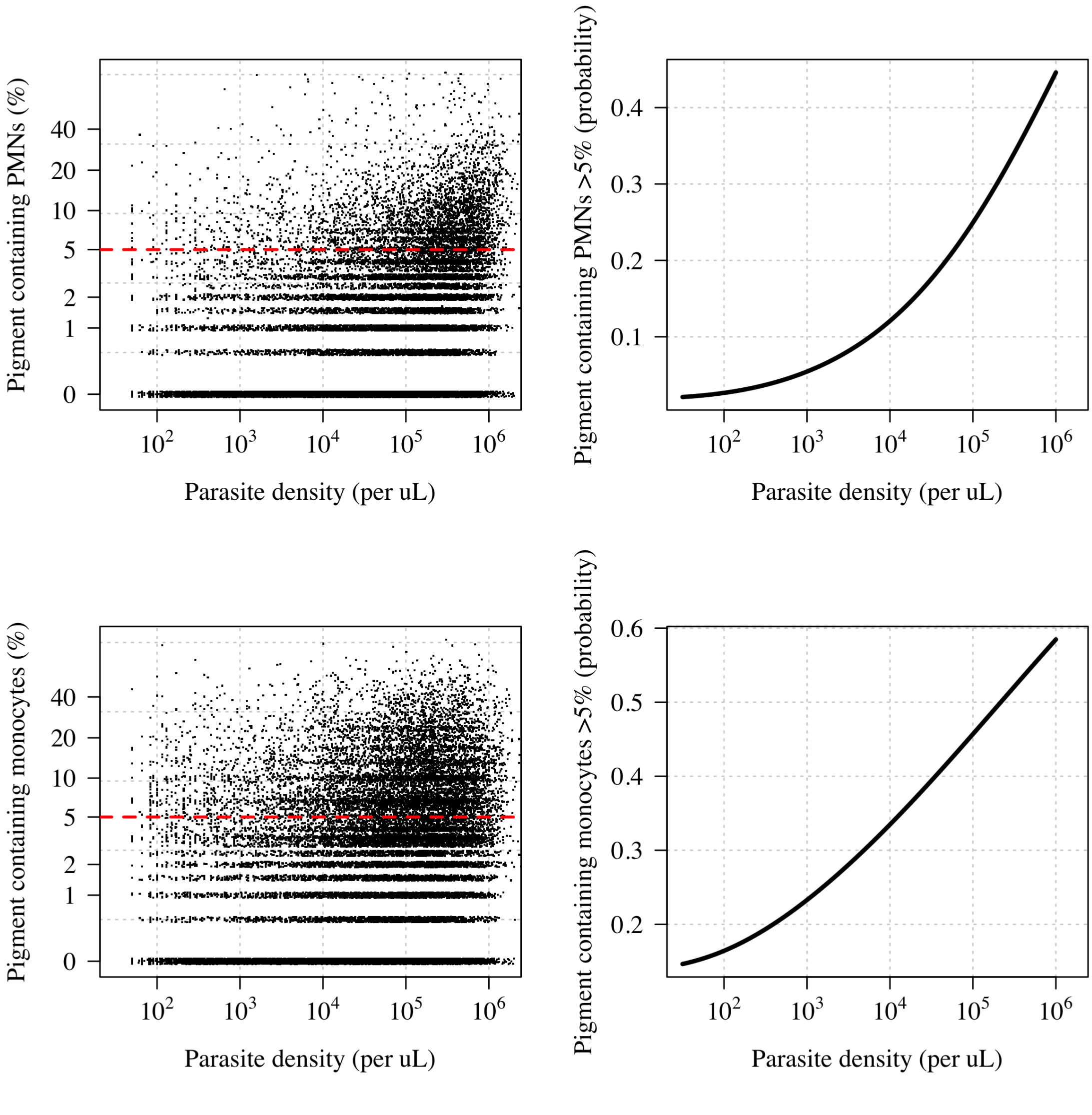

Figure S7: Relationship between parasite density and intraleukocytic malaria pigment (top: PMNs, bottom: monocytes). The left panels show scatterplots for the whole pooled data set; the right panels show the probability of having >5% pigment containing PMNs (neutrophils) or monocytes as a function of the peripheral blood malaria parasite density (fitted using a logistic regression model with nested random effects for site, country and study).

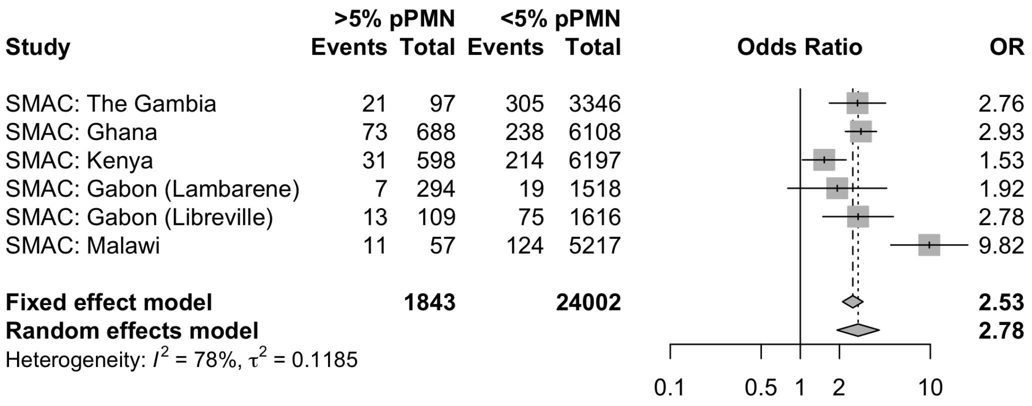

Figure S8: Prognostic value for mortality of >5% versus ≤ 5% pigment containing PMNs (neutrophils) in patients from the SMAC study. The individual estimates (vertical ticks) and 95% confidence intervals (horizontal lines) are shown broken down by country.

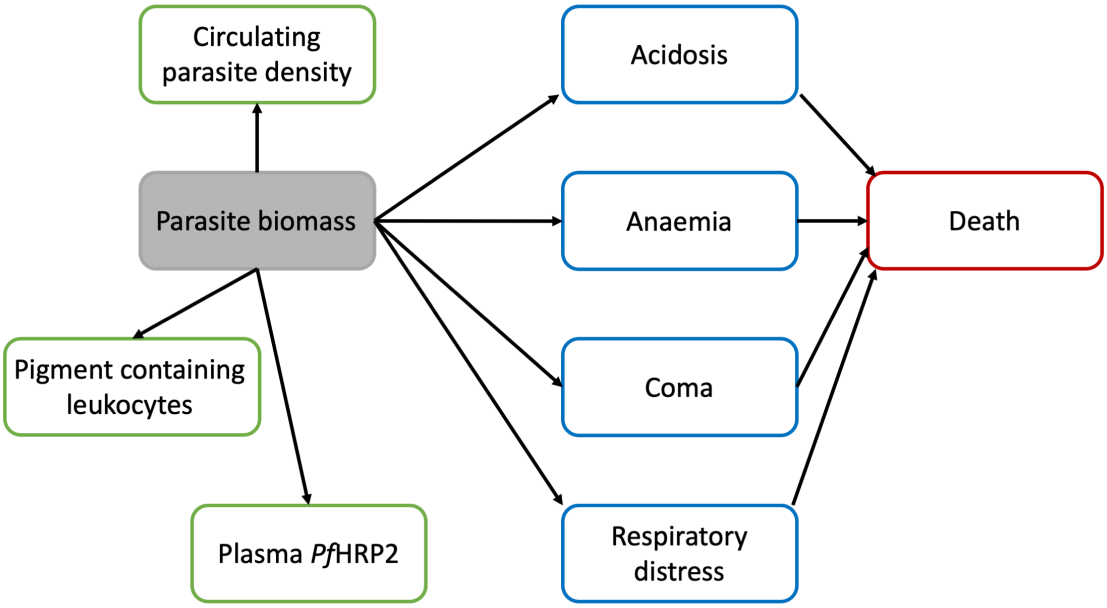

Figure S9: Proposed causal diagram for the relationship between intraleukocytic pigment counts, plasma *Pf*HRP2, parasite counts, and mortality in severe malaria. The main determinant of mortality is the parasite biomass which is unobservable (grey). The three exposure variables of interest (green) are proxy measurements of varying accuracy for the parasite biomass. The clinical variables in blue mediate the relationship between the parasite biomass and death (red).

Prognostic factors in severe malaria

These are estimated from our pooled data set, looking at African children only (n=31538), in which the aggregate mortality was 5.3% (1678/31538). We report point estimates from the 2x2 table (ignoring differences between sites and studies and simply counting the number of deaths in each category. In this dataset the odds-ratio for death in patients with >5% pigment containing PMNs was 2.5 (10.5% versus 4.4% mortality; 1186/26766 versus 362/3456).

For reference, we estimate in the same dataset the odds-ratio for death for other key indicators:

- Coma: odds ratio is 7.6 (19.3% versus 3.% mortality; 825/27115 versus 851/4410);
- Respiratory distress (Kussmaul's breathing): odds-ratio is 6.6 (19.0% versus 3.4%; 713/3750 versus 950/27616);
- Laboratory defined acidosis (lactate>5 mmol or BUN>10 mg/dL): odds ratio is 4.9 (11.1% versus 2.5% mortality; 1019/9210 versus 489/19842);
- Severe anaemia (haemoglobin < 5g/dL): odds ratio is 1.8 (7.7% versus 4.5% mortality; 530/6922 versus 1057/23689);
- Hyperparasitaemia (>100,000 parasites per μL): odds ratio is 1 (5.2% versus 5.2% mortality; 645/12323 versus 958/18545);

Systematic review

The systematic review identified 19 relevant publications which described clinical series in which pigment containing peripheral blood leukocytes had been counted. In most the the relationship to the severity of malaria (or death) had been evaluated [4–22]. The majority of these studies had very small sample sizes relative to the AQUAMAT and SEAQUAMAT randomised controlled trials (the median sample size was 67 for the number of severe malaria patients; some studies included uncomplicated malaria). We decided to contact study authors to obtain individual patient data for studies with at least 100 severe malaria patients. The data from Boeuf *et al* [16] could not be obtained as the principal investigator who had access to the data was deceased. The data from Luty *et al* could not be obtained as the authors could not find the original data. The data from Birhanu could not be obtained as the contact details were out of date and the corresponding author could not be contacted.

Previous reports

1. Phu *et al* (1995) described a prospective series of 300 consecutive adult Vietnamese patients with severe falciparum malaria (AQ Vietnam trial). The 40 who died had significantly higher proportions of malaria pigment-containing neutrophils on admission (mean=7.7%, standard deviation (SD)=5.9%) and pigment-containing monocytes (mean=8.6%, SD=5.9%) than did survivors (mean 3.2%, SD=4.1% and mean 4.8% (SD 4.6%) respectively) (P<0.0001). A count of peripheral neutrophils containing visible pigment >5% predicted a fatal outcome with 73% sensitivity and 77% specificity (relative risk 6.2, 95% confidence interval (CI) 3.2-11.8) compared to 60% sensitivity and 57% specificity for parasitaemia >100,000/uL (relative risk 1.8, 95% CI 1.0-3.3).

2. Metzger *et al* (1995) reported a series from Gabon comprising 42 children with severe malaria, 31 children with "mild" malaria, and 31 adults with "mild" malaria. More pigment-containing monocytes and pigment-containing neutrophils were found in severe malaria. There was one death (11% pigment containing neutrophils). The authors concluded that the elevation in pigment-containing monocytes was more informative.

3. Amodu *et al* (1998) described a series of 146 Nigerian children aged between 6 months and 14 years and divided them in four categories, of whom 43 had severe malaria. The median proportion of pigment-containing neutrophils showed a clear rise across the spectrum from no malaria (2.0%), asymptomatic malaria (6.5%), mild malaria (9.0%) and cerebral malaria (27.0%) (P < 0.0001). The proportion of pigment-containing monocytes did not

differ significantly between the mild malaria, asymptomatic malaria and no malaria groups but the cerebral malaria group had a higher median value than the other three groups.

4. Luty *et al* (2000) conducted a case-control comparison of 100 patients with severe malaria (cases) and 100 patients with mild malaria (controls). The proportions of pigment-containing monocytes and pigment-containing neutrophils on admission were positively correlated with parasitaemia and were 4- and 18-fold-larger respectively in severe compared with mild malaria

5. Lyke *et al* (2003) studied 172 Malian children with severe malaria and 172 controls with uncomplicated malaria. The presence of polymorphonuclear cell (PMN) and monocyte (PMM) pigment was strongly associated with severe disease compared with uncomplicated malaria. Total PMN pigment burden in children with severe malaria was higher in those with cerebral manifestations and with combined cerebral manifestations and severe anaemia (haemoglobin <5 g/dL) but was not associated with hyperparasitaemia ($>$ 500,000 asexual forms/mm3). Additionally, the count of pigment containing PMNs was associated with a fatal outcome in patients with severe malaria.

6. Ochola *et al* (2005) studied prospectively 22 Kenyan children with severe malaria. Peripheral parasitaemia, plasma levels of sTNF-R75 and circulating parasite DNA correlated better with estimated sequestered parasite load than pigment containing leukocytes.

7. Mujuzi *et al* (2006) conducted a case-control study of infants with severe malaria in Uganda; 99 with severe malaria and 96 with uncomplicated malaria. Higher proportions of pigment containing monocytes were associated with anaemia.

8. Casals-Pascual *et al* (2006) studied 26 Kenyan children with severe malarial anaemia. Haemozoin-containing neutrophils were strongly associated with plasma lactate levels (a well characterized prognostic measure in severe malaria), independently of parasite density ($r = 0.60$, $P = 0.003$).

9. Hanscheid *et al* (2008) studied 152 patients in Gabon with falciparum malaria. Compared with the 104 children with uncomplicated malaria the 48 children with severe malaria had higher proportions of pigment-containing neutrophils but not monocytes.

10. Kremsner *et al* (2009) analysed pigment containing leukocytes in the admission thick blood smears of 26,296 children hospitalized with P. falciparum and studied in the Severe Malaria in African Children network. They found that at all but one of the study sites the mortality for patients with >5 pigment containing granulocytes per 200 white blood cells was higher than in subjects with no pigment containing granulocytes. However, they described adjusted odds ratios for associations between mortality and pigment containing leukocytes, estimated through logistic regression and which included other established markers of severe malaria, as "moderate to nonexistent in most sites". They concluded that "levels of pigmented cells were not useful predictors of outcome across Africa".

11. Novelli *et al* (2010) studied 355 Kenya children falciparum malaria, into three groups: uncomplicated malaria (Hb >11.0 g/dL, n=23); anaemic (Hb 6-10.9g/dL, n=200), and severe malarial anaemia (Hb $<$ 6.0 d/dL, n=132). In a multiple logistic regression analysis pigment containing monocytes were associated independently with severe malarial anaemia. There were no deaths in the severe malaria group.

12. Fendel *et al* (2010) studied a series of children with severe malarial anaemia in Gabon. The proportions of pigment containing monocytes were higher in the 39 children with severe anaemia compared with values in the 52 with mild malaria.

13. Boeuf *et al* (2012) conducted a prospective study of Ghanaian children with falciparum mlaria; 144 had cerebral malaria, 108 severe anaemia and 80 had uncomplicated malaria. The proportions of pigment-containing monocytes were higher in cerebral malaria than in severe anaemia, and both were higher than in uncomplicated malaria.

14. Abugri *et al* (2014) studied 100 children with falciparum malaria in Ghana. The proportions of pigment-containing monocytes were higher in severe malaria (n=32) than they were in uncomplicated malaria (n=68).

15. Kempaiah (2016) studied 144 children with falciparum malaria, stratified into two clinical categories: non-SMA (Hb >5.0 g/dL, *n*=91) and SMA (Hb <5.0 g/dL, *n*=53). They showed that people with severe anaemia (Hb<5) had a lower haemoglobin than people without severe anaemia (Hb>5), p<0.0001.

16. Mandala (2016) enrolled children presenting with cerebral malaria (*n*=36), severe malarial anaemia (*n*=42) or uncomplicated malaria (*n*=66), and healthy aparasitemic children (*n*=52) in Blantyre, Malawi. They observed that in all malaria groups, especially the severe malarial anaemia group, a greater proportion of monocytes contained haemozoin pigment compared to controls.

17. Birhanu *et al* (2017) studied children hospitalized with malaria in Ethiopia, 283 of whom had *P. falciparum* infections, 57 had *P. vivax* and 37 were mixed. Of these 102 children had severe malaria. Both elevated pigment-containing monocytes and pigment-containing neutrophils were associated independently with severe malaria (AOR = 6.26, 95% CI: 2.14–14.29 and AOR = 7.93, 95% CI: 3.09–16.86 respectively).

18. Salih *et al* (2018) conducted a case control study in Sudanese children; 67 with severe falciparum malaria, 63 with uncomplicated falciparum malaria and 50 healthy children. Haemozoin containing monocytes were more common in severe malaria than in uncomplicated malaria.

19. Achieng *et al* (2019) compared 53 parasitaemic Kenyan children with severe malaria anaemia SMA (Hb<5.0g/dL) and 69 parasitaemic children with higher haemoglobin values. Counts of pigment-containing monocytes and pigment-containing neutrophils were higher in children severe malaria anaemia (P=.028 and P=.079, respectively).

The study of Davenport *et al* (2010) is not included in our list as this concentrated on the impact of HIV infection on clinical severity and longer term prognosis. This was a study of 542 Kenyan children aged 3-36 months with falciparum malaria. HIV positive children had significantly more malarial pigment-containing neutrophils, monocytosis, and were more likely to be severely anaemic increased (Hb < 6.0 g/dL), and were nearly ten times more likely to die within 3 months of enrollment. Increased proportions of pigment-containing monocytes but not pigment-containing neutrophils were associated significantly with severe malarial anaemia. Patients with any density of *P. falciparum* were included in the study. None of the children had cerebral malaria and it is unclear how many would have met the WHO definition for severe malarial anaemia.

Data collection from included studies

For all studies that passed screening and had more than 100 patients with severe malaria, we contacted the corresponding author using available emails addresses found online. For the SMAC study, the data had been deposited open access (https://dataverse.harvard.edu/dataset.xhtml?persistentId=doi:10.7910/DVN/OCTWUJ) but we checked our findings with the corresponding author. One author could not be contacted (Birhanu et al).

Data requested for each study included all intraleukocytic pigment counts (number of cells counted and number containing pigment), the parasite count at baseline, in-hospital mortality outcome, and any available baseline clinical or laboratory variables. For each included dataset we plotted all relevant data to check integrity and data quality. R scripts used to clean the SMAC study data are available on the linked github repository (https://github.com/jwatowatson/MalariaPigmentPrognosis).

Risk of bias and applicability using a modified QUADAS-2 criteria tool

In all five studies, the patients were hospital cohorts (no risk of bias in how they were selected). In all studies microscopy was done blinded to patient allocation (randomised trials) and outcome. For all trials the outcome was in-hospital mortality (very low risk of bias).

References

[1] Tran Tinh Hien et al. "A controlled trial of artemether or quinine in Vietnamese adults with severe falciparum malaria". In: *New England Journal of Medicine* 335.2 (1996), pp. 76–83.

	Patient selection (risk of bias)	Microscopy evaluation (risk of bias)	Outcome evaluation (risk of bias)	Patient selection (applicability)	Outcome evaluation (applicability)
AQ Vietnam [1]	LOW	LOW	LOW	LOW	HIGH
SEAQUAMAT [2]	LOW	LOW	LOW	LOW	HIGH
AQUAMAT [3]	LOW	LOW	LOW	MODERATE	HIGH
Lyke et al [5]	LOW	LOW	LOW	MODERATE	HIGH
SMAC [4]	LOW	LOW	LOW	MODERATE	HIGH

Table S2: Assessment of the risk of bias and applicability of the included studies using a modified QUADAS-2 tool [23].

[2] Arjen M Dondorp et al. "Artesunate versus quinine for treatment of severe falciparum malaria: a randomised trial". In: *The Lancet* 366.9487 (2005), pp. 717–725.

[3] Arjen M Dondorp et al. "Artesunate versus quinine in the treatment of severe falciparum malaria in African children (AQUAMAT): an open-label, randomised trial". In: *The Lancet* 376.9753 (2010), pp. 1647–1657.

[4] Peter Gottfried Kremsner et al. "Prognostic value of circulating pigmented cells in African children with malaria". In: *The Journal of Infectious Diseases* 199.1 (2009), pp. 142–150.

[5] Kirsten E Lyke et al. "Association of intraleukocytic *Plasmodium falciparum* malaria pigment with disease severity, clinical manifestations, and prognosis in severe malaria". In: *The American Journal of Tropical Medicine and Hygiene* 69.3 (2003), pp. 253–259.

[6] Nguyen Hoan Phu et al. "Intraleucocytic malaria pigment and prognosis in severe malaria". In: *Transactions of The Royal Society of Tropical Medicine and Hygiene* 89.2 (Mar. 1995), pp. 200–204. ISSN: 0035-9203.

[7] Wolfram G Metzger, Benjamin G Mordmüller, and Peter G Kremsner. "Malaria pigment in leucocytes". In: *Transactions of the Royal Society of Tropical Medicine and Hygiene* 89.6 (1995), pp. 637–638.

[8] Olukemi K Amodu et al. "Intraleucocytic malaria pigment and clinical severity of malaria in children". In: *Transactions of the Royal Society of Tropical Medicine and Hygiene* 92.1 (1998), pp. 54–56.

[9] Adrian J. F. Luty et al. "Low Interleukin-12 Activity in Severe *Plasmodium falciparum* Malaria". In: *Infection and Immunity* 68.7 (2000), pp. 3909–3915.

[10] LB Ochola et al. "Estimation of the sequestered parasite load in severe malaria patients using both host and parasite markers". In: *Parasitology* 131.4 (2005), pp. 449–458.

[11] Godfrey Mujuzi et al. "Pigmented monocytes are negative correlates of protection against severe and complicated malaria in Ugandan children". In: *American Journal of Tropical Medicine and Hygiene* 74.5 (2006), pp. 724–729.

[12] Climent Casals-Pascual et al. "Lactate levels in severe malarial anaemia are associated with haemozoin-containing neutrophils and low levels of IL-12". In: *Malaria Journal* 5.1 (Nov. 2006), p. 101.

[13] Thomas Hänscheid et al. "Full blood count and haemozoin-containing leukocytes in children with malaria: diagnostic value and association with disease severity". In: *Malaria Journal* 7.1 (2008), pp. 1–10.

[14] Enrico M. Novelli et al. "Clinical predictors of severe malarial anaemia in a holoendemic Plasmodium falciparum transmission area". In: *British Journal of Haematology* 149.5 (2010), pp. 711–721.

[15] Rolf Fendel et al. "Hemolysis is associated with low reticulocyte production index and predicts blood transfusion in severe malarial anemia". In: *PloS one* 5.4 (2010), e10038.

[16] Philippe S Boeuf et al. “Insights into deregulated TNF and IL-10 production in malaria: implications for understanding severe malarial anaemia”. In: *Malaria Journal* 11.1 (2012), pp. 1–9.

[17] James Abugri et al. “Age-related pattern and monocyte-acquired haemozoin associated production of erythropoietin in children with severe malarial anaemia in Ghana”. In: *BMC Research Notes* 7.1 (2014), pp. 1–6.

[18] Prakasha Kempaiah et al. “Reduced Hsp70 and Glutamine in Pediatric Severe Malaria Anemia: Role of hemozoin in Suppressing Hsp70 and NF-κB Activation”. In: *Molecular Medicine* 22.1 (Jan. 2016), pp. 570–584.

[19] W. L. Mandala et al. “Monocyte activation and cytokine production in Malawian children presenting with P. falciparum malaria”. In: *Parasite Immunology* 38.5 (2016), pp. 317–325.

[20] Muluken Birhanu et al. “Hematological Parameters and Hemozoin-Containing Leukocytes and Their Association with Disease Severity among Malaria Infected Children: A Cross-Sectional Study at Pawe General Hospital, Northwest Ethiopia”. In: *Interdisciplinary Perspectives on Infectious Diseases* 2017 (Feb. 2017), p. 8965729.

[21] Magdi M. Salih et al. “Haematological parameters, haemozoin-containing leukocytes in Sudanese children with severe Plasmodium falciparum malaria”. In: *The Journal of Infection in Developing Countries* 12.04 (Apr. 2018).

[22] Angela O. Achieng et al. “Molecular basis of reduced LAIR1 expression in childhood severe malarial anaemia: Implications for leukocyte inhibitory signalling”. In: *EBioMedicine* 45 (2019), pp. 278–289.

[23] Penny F Whiting et al. “QUADAS-2: A Revised Tool for the Quality Assessment of Diagnostic Accuracy Studies”. In: *Annals of Internal Medicine* 155.8 (2011), pp. 529–536.
