# Supplementary material for: The prognostic and diagnostic value of intraleukocytic malaria pigment in patients with severe falciparum malaria
Source: Nat Commun. 2022 Nov 12;13:6882. doi: 10.1038/s41467-022-34678-8 (PMC9653500; doi:10.1038/s41467-022-34678-8)
Supplement: Supplementary file 3 — Reporting Summary [file 41467_2022_34678_MOESM3_ESM.pdf]

nature portfolio

Corresponding author(s): James A Watson

Last updated by author(s): Oct 26, 2022

Reporting Summary

Nature Portfolio wishes to improve the reproducibility of the work that we publish. This form provides structure for consistency and transparency in reporting. For further information on Nature Portfolio policies, see our Editorial Policies and the Editorial Policy Checklist.

Statistics

For all statistical analyses, confirm that the following items are present in the figure legend, table legend, main text, or Methods section.

n/a	Confirmed	
☐	☒	The exact sample size (n) for each experimental group/condition, given as a discrete number and unit of measurement
☐	☒	A statement on whether measurements were taken from distinct samples or whether the same sample was measured repeatedly
☐	☒	The statistical test(s) used AND whether they are one- or two-sided<br>*Only common tests should be described solely by name; describe more complex techniques in the Methods section.*
☐	☒	A description of all covariates tested
☐	☒	A description of any assumptions or corrections, such as tests of normality and adjustment for multiple comparisons
☐	☒	A full description of the statistical parameters including central tendency (e.g. means) or other basic estimates (e.g. regression coefficient) AND variation (e.g. standard deviation) or associated estimates of uncertainty (e.g. confidence intervals)
☐	☒	For null hypothesis testing, the test statistic (e.g. F, t, r) with confidence intervals, effect sizes, degrees of freedom and P value noted<br>*Give P values as exact values whenever suitable.*
☒	☐	For Bayesian analysis, information on the choice of priors and Markov chain Monte Carlo settings
☒	☐	For hierarchical and complex designs, identification of the appropriate level for tests and full reporting of outcomes
☐	☒	Estimates of effect sizes (e.g. Cohen's d, Pearson's r), indicating how they were calculated

Our web collection on statistics for biologists contains articles on many of the points above.

Software and code

Policy information about availability of computer code

Data collection	no software was used for data collection
Data analysis	All data analysis was done using R version 4.0.2 using the following packages: mgcv version 1.8; lme4 version 1.1; meta version 4.19; bootpredictlme4 version 0.1. All code is available at github.com/jwatowatson/MalariaPigmentPrognosis

For manuscripts utilizing custom algorithms or software that are central to the research but not yet described in published literature, software must be made available to editors and reviewers. We strongly encourage code deposition in a community repository (e.g. GitHub). See the Nature Portfolio guidelines for submitting code & software for further information.

Data

Policy information about availability of data

All manuscripts must include a data availability statement. This statement should provide the following information, where applicable:

- Accession codes, unique identifiers, or web links for publicly available datasets
- A description of any restrictions on data availability
- For clinical datasets or third party data, please ensure that the statement adheres to our policy

Pigment containing PMN counts and pigmented PMM counts for the AQ Vietnam, SEAQUAMAT and AQUAMAT trials and the Lyke et al and SMAC studies are available along with parasite counts, coma, acidosis and outcome on the github repository github.com/jwatowatson/MalariaPigmentPrognosis. This has been archived on Zenodo with the following DOI: 10.5281/zenodo.5720162.
Data from the SMAC network are openly available at: https://dataverse.harvard.edu/dataset.xhtml?persistentId=doi:10.7910/DVN/0CTWUJ

Field-specific reporting

Please select the one below that is the best fit for your research. If you are not sure, read the appropriate sections before making your selection.

☒ Life sciences ☐ Behavioural & social sciences ☐ Ecological, evolutionary & environmental sciences

For a reference copy of the document with all sections, see nature.com/documents/nr-reporting-summary-flat.pdf

Life sciences study design

All studies must disclose on these points even when the disclosure is negative.

Sample size	No sample size calculation was performed. We analysed all available data.
Data exclusions	No data were excluded.
Replication	No experiments were replicated
Randomization	Not a randomised study. The exposure of interest is the proportion of the pigment counts. In the main analysis no covariates were controlled for as we were interested in the prognostic value of the pigment counts on their own.
Blinding	Microscopists were blinded to treatment allocation and patient severity.

Reporting for specific materials, systems and methods

We require information from authors about some types of materials, experimental systems and methods used in many studies. Here, indicate whether each material, system or method listed is relevant to your study. If you are not sure if a list item applies to your research, read the appropriate section before selecting a response.

Materials & experimental systems

n/a	Involved in the study	
☒	☐	Antibodies
☒	☐	Eukaryotic cell lines
☒	☐	Palaeontology and archaeology
☒	☐	Animals and other organisms
☐	☒	Human research participants
☐	☒	Clinical data
☒	☐	Dual use research of concern

Methods

n/a	Involved in the study	
☒	☐	ChIP-seq
☒	☐	Flow cytometry
☒	☐	MRI-based neuroimaging

Human research participants

Policy information about studies involving human research participants

Population characteristics	All participants are severely ill adults or children who were clinically diagnosed with severe falciparum malaria. In the African studies the large majority are children under the age of 14, in the Asian studies the majority are adults.<br>The mean age was 4.5 years (range: 0 to 87 years). Sex was not recorded in the merged dataset.
Recruitment	All patients were enrolled in large hospitals that routinely treat severe malaria. All patients who were willing to consent to trial or study procedures and who met the inclusion criteria were enrolled. No selection biases are of concern.
Ethics oversight	The trial protocols were reviewed and approved by each site's appropriate ethical review board (ERBs), and also by the Oxford Tropical Research Ethics committee (OXTREC). Re-use of existing, appropriately anonymized, human data does not require ethical approval under the Oxford Tropical Research Ethics Committee regulations (OxTREC).

Note that full information on the approval of the study protocol must also be provided in the manuscript.

Clinical data

Policy information about clinical studies

All manuscripts should comply with the ICMJE guidelines for publication of clinical research and a completed CONSORT checklist must be included with all submissions.

Clinical trial registration	The AQ Vietnam trial and the SEAQUAMAT trial were not registered as they were done before 2005. The AQUAMAT trial: ISRCTN50258054

Study protocol	Contact the study authors
Data collection	AQ Vietnam: conducted between May 1991 and January 1996 in a specialist ward of the Hospital for Tropical Diseases, Ho Chi Minh City.<br>SEAQUAMAT: The participating centres were located in Bangladesh, Myanmar (7 hospitals), India, and Indonesia June, 2003, and May, 2005<br>AQUAMAT: conducted between Oct 3, 2005, and July 14, 2010 in 11 centres in nine countries (Mozambique, The Gambia, Ghana, Kenya, Tanzania, Nigeria, Uganda, Rwanda, and Democratic Republic of the Congo) in Africa
Outcomes	The pre-specified primary outcome in all three randomised trials was in-hospital mortality (discharged alive is survival). This was also pre-specified for the meta-analysis in the PROSPERO registration document (CRD42021284527).
